# Alterations in gene expression profiles correlated with cisplatin cytotoxicity in the glioma U343 cell line

**DOI:** 10.1590/S1415-47572010005000013

**Published:** 2010-03-01

**Authors:** Patricia Oliveira Carminati, Stephano Spano Mello, Ana Lucia Fachin, Cristina Moraes Junta, Paula Sandrin-Garcia, Carlos Gilberto Carlotti, Eduardo Antonio Donadi, Geraldo Aleixo Silva Passos, Elza Tiemi Sakamoto-Hojo

**Affiliations:** 1 Departamento de Genética, Faculdade de Medicina de Ribeirão Preto, Universidade de São Paulo, SPBrazil; 2 Departamento de Cirurgia e Anatomia, Faculdade de Medicina de Ribeirão Preto, Universidade de São Paulo, Ribeirão Preto, SPBrazil; 3 Departamento de Clínica Médica, Faculdade de Medicina de Ribeirão Preto, Universidade de São Paulo, Ribeirão Preto, SPBrazil; 4 Faculdade de Odontologia de Ribeirão Preto, Universidade de São Paulo, Ribeirão Preto, SPBrazil; 5 Departamento de Biologia, Faculdade de Filosofia, Ciências e Letras de Ribeirão Preto, Universidade de São Paulo, Ribeirão Preto, SPBrazil

**Keywords:** apoptosis, cisplatin, gene expression, glioma

## Abstract

Gliomas are the most common tumors in the central nervous system, the average survival time of patients with glioblastoma multiforme being about 1 year from diagnosis, in spite of harsh therapy. Aiming to study the transcriptional profiles displayed by glioma cells undergoing cisplatin treatment, gene expression analysis was performed by the cDNA microarray method. Cell survival and apoptosis induction following treatment were also evaluated. Drug concentrations of 12.5 to 300 μM caused a pronounced reduction in cell survival rates five days after treatment, whereas concentrations higher than 25 μM were effective in reducing the survival rates to ~1%. However, the maximum apoptosis frequency was 20.4% for 25 μM cisplatin in cells analyzed at 72 h, indicating that apoptosis is not the only kind of cell death induced by cisplatin. An analysis of gene expression revealed 67 significantly (FDR < 0.05) modulated genes: 29 of which down- and 38 up-regulated. These genes belong to several classes (metabolism, protein localization, cell proliferation, apoptosis, adhesion, stress response, cell cycle and DNA repair) that may represent several affected cell processes under the influence of cisplatin treatment. The expression pattern of three genes (*RHOA*, *LIMK2* and *TIMP2*) was confirmed by the real time PCR method.

## Introduction

Malignant gliomas are the most common primary malignancies in the brain, comprising more than 60% of primary brain tumors (Huang *et al.*, 2002; Iwadate *et al.*, 1996; Kunwar *et al.*, 2001). In an adult population, this type of tumor accounts for about 1% of all cancers, with more than 2% of deaths being attributed to malignant gliomas (Wong *et al.*, 2007). The average survival time of patients with the most malignant type, glioblastoma multiforme, is about 1 year after diagnosis, irrespective of the aggressive combination of surgery, radiotherapy and chemotherapy. Prognosis, in the case of malignant astrocytic gliomas, is dismal, due to their ability to diffusely infiltrate into the normal brain parenchyma. In cell cultures, malignant glioma cells proved to be very resistant to apoptosis induced by various anticancer agents (Bogler and Weller, 2002; Iwamaru *et al.*, 2007; Lefranc *et al.*, 2005). In spite of advances in anticancer therapies, the prognosis for glioma patients is still very discouraging (Ohgaki, 2005).

Cisplatin is a DNA-damaging agent used in first-line chemotherapy against epithelial malignancies of the lungs, ovaries, bladder, testis, head, neck, esophagus, gut, colon and pancreas, as also in second- and third-line treatment against a number of metastatic malignancies, including breast and prostate cancer, melanomas, malignant gliomas and others (Boulikas and Vougiouka, 2004). Cisplatin forms primarily 1,2-intrastrand crosslinks between adjacent purines in DNA and also introduces DNA 1,3-intrastrand crosslinks and, to a lesser extent, interstrand crosslinks. The mechanisms of cisplatin-induced cytotoxicity are not completely understood yet. However, it has been reported that the antitumoral activity of cisplatin is probably due to the formation of DNA adducts that block DNA replication and transcription, thereby triggering cellular responses, including apoptosis (Brabec and Kasparkova, 2005; Torigoe *et al.*, 2005; Zhang *et al.*, 2006).

The development of cDNA microarrays technology has facilitated the analysis of gene expression profiles that can generate a large body of information on genes and pathways related to the response to several antitumoral drugs (Li *et al.*, 2007).

In order to investigate how glioma cells respond to antitumoral cisplatin, we measured cell survival and apoptosis induction, in addition to analysis of gene expression displayed by cisplatin-treated compared with untreated U343 cells, by using the cDNA microarray technique. This approach was propitious for registering significantly modulated genes that play important roles in the innumerous signaling pathways involved in cisplatin-treated glioma cells. While providing a general characterization of cisplatin cytotoxicity in U343 cells, we showed that at conditions of moderate to high drug cytotoxicity (25 μM cisplatin), capable of inducing a significant reduction in survival rates after 5 days of treatment, transcriptional changes involved the modulation of several genes belonging to diverse functional categories. The main biological processes associated with these modulated genes were metabolism, cell proliferation, apoptosis, cell adhesion, stress response, cell cycle control and DNA repair.

## Material and Methods

###  Cell culture conditions and reagents

Human glioma cell line U343 was kindly provided by Dr. James T. Rutka (The Arthur and Sonia Labatt Brain Tumour Research Center, Canada). MRC-5 (SV-40 transformed fibroblast cell line) was provided by Dr. Carlos F. M. Menck (ICB-USP, São Paulo, Brazil). Cells were routinely grown in Dulbecco's modified Eagle's medium (DMEM) + F10 (1:1) (Sigma Aldrich, St. Louis, MO, USA), supplemented with 15% fetal bovine serum (Cultilab, Campinas, SP, Brazil), ciprofloxacin and kanamicin in 25 cm^2^ culture flasks (Corning, NY, USA). The cell cultures were kept at 37 °C in a humidified atmosphere of 5% CO_2_. Cisplatin (Sigma Aldrich, St. Louis, MO, USA) was dissolved in sterile water just before use.

###  Cell survival

Cells were treated with 12.5; 25; 50; 75; 150 and 300 μM cisplatin and harvested at 24 h and 5 days later. Cell survival after cisplatin treatment was measured by using the Cell Proliferation Kit II (Roche) containing the tetrazolium salt XTT. Surviving cells with active mitochondria are capable of cleaving the XTT substrate into an orange formazan dye. After 1 h incubation, the amount of formazan dye can be measured by spectrophotometry (Amersham Biosciences, England, UK) analysis performed at optical densities (OD) of 492 and 690 nm. Cell survival was calculated as the percentage of absorbance displayed by cisplatin treated cells compared to untreated cells. Each experiment was repeated at least three times.

###  Apoptosis detection

Cisplatin-induced apoptosis was determined using a mixture of propidium iodide (5 μg/mL), fluorescein diacetate (15 μg/mL) and Hoechst 33342 (2 μg/mL) (all from Sigma Aldrich, St. Louis, MO, USA). Cells treated with several concentrations of cisplatin (12.5; 25 and 50 μM) were harvested at different times (24, 48 and 72 h) after treatment. Floating and adherent cells were collected and stained. 500 cells per treatment were examined through a fluorescence microscope (Axiophot, Zeiss) to score apoptotic cells. At least three independent experiments were carried out.

###  RNA extraction and gene expression

Experiments for gene expression analysis were carried out on U343 cells treated with 25 μM cisplatin for 48 h. A total of four independent experiments were made, and RNA extraction was performed at 48 h following cisplatin treatment. Total RNA was isolated from cultured cells using the Trizol reagent according to manufacturer's instructions. The integrity of RNA samples was evaluated by denaturing agarose gel electrophoresis under standard conditions.

###  cDNA microarrays

For gene expression analysis, 936 cDNA clones from the IMAGE consortium were used to construct the microarray on nylon membrane. These clones, kindly provided by Dr Catherine Nguyen (INSERM, Marseille, France), were amplified on 96-well plates by polymerase chain reaction (PCR). The PCR products were purified and spotted in duplicate onto Hybond N+ membranes (Amersham Pharmacia Biotech, Buckinghamshire, UK), using the Generation III Microarray Spotter device (Amersham Pharmacia Biotech, Buckinghamshire, UK).

###  cDNA probe labeling and hybridization

Hybridization was carried out with a ^33^P-labeled oligonucleotide, using the T4 kinase labeling kit (Invitrogen, Carlsbad, CA). Membranes were pre-hybridized in a hybridization mix (5x SSC, 5x Denhardt's solution, 0.5% SDS and 100 μg/mL of salmon sperm DNA) at 42 °C for 24 h, followed by hybridization with the vector probe 1S at 42 °C for 24 h. Membranes were washed with 2x SSC, 0.1% SDS solution for 10 min at room temperature, and exposed to radiation-sensitive imaging plates for 24 h. Hybridization signals were detected in a phosphor image device (Cyclone, Packard Instruments, USA).

The complex probe was prepared with 10 μg of total RNA and 8 μg of oligo(dT)25. The reaction mixture was incubated for 8 min at 70 °C, and then cooled to 42 °C. This process improves long polyA tail saturation. Reverse transcription was performed in a reaction mixture containing 1 μL of RNasin (Promega, 40U/ul), 6 μL of buffer 5x, 2 μL of DTT 0.1 M, 0.6 μL of dATP 20 mM, 0.6 μL of dTTP 20 mM, 0.6 μL of dGTP 20 mM, 0.6 μL of dCTP 120 μM, 3 μL of α ^33^P-dCTP, 1 μL of reverse transcriptase (SUPERSCRIPT RNase H free RT, Invitrogen, 200 U/μL), and 2.8 μL of sterile water. After 1 h at 42 °C, 1 μL of reverse transcriptase was added and the mix was incubated for 1 h at 42 °C. Subsequently, 1 μL of SDS 10%, 1 μL of EDTA 0.5 M and 3 μL of NaOH 3 M were added to the mixture in order to degrade mRNA and rRNA templates. The reaction mixture was incubated for 30 min at 68 °C and then for 15 min at room temperature. Finally, 10 μL of Tris 1 M, 3 μL of HCl 2N were added to neutralize the reaction. The volume was completed to 100 μL, and the probe purified on a Sephadex G-50 column.

Membranes were placed into hybridization flasks, and hybridization with the complex probe was performed at 65 °C for 48 h, followed by washes with 0.1x SSC, 0.1% SDS at 68 °C for 3 h, and exposure to radiation-sensitive imaging plates for 48 h. Images were captured in a phosphor image device (Cyclone, Packard Instruments, USA). Thereafter, numerical values obtained for hybridization signals were quantified by using the BZScan software (Rougemont and Hingamp, 2003).

###  Analysis of microarray data

Data obtained by using the BZScan software were normalized through the following steps: background subtraction; normalization of the amount of spotted cDNA by oligo-vector labeling values and correlation-based filtering of array elements, which indicated unreliable elements with low correlation. A global normalization procedure was performed, which consisted of dividing all the individual spot values obtained in one experiment by the median value calculated for the whole experiment (Quackenbush, 2002). The normalized data were analyzed by MEV software. Statistical analysis by t-test and SAM (Significance Analysis of Microarrays) method (Tusher *et al.*, 2001) were performed in order to select significantly modulated genes at a FDR (False Discovery Rate) < 0.05. In order to search for gene functions, the data were submitted to S.O.U.R.C.E. (Stanford Online Universal Resource for Clones and ESTs), NCBI and DAVID-NIH (Dennis *et al.*, 2003). For three genes, the transcriptional profiles were confirmed by the real-time PCR method.

###  Real time PCR method

A quantitative real time PCR (qPCR) method was used to confirm gene expression profiles for three genes, *TIMP2*, *RHOA* and *LIMK2*. RNA samples used in cDNA microarrays were submitted to decontamination of DNA traces by the treatment with the Deoxyribonuclease I, Amplification Grade kit (Invitrogen), according to manufacturer's instructions. The reverse transcription step was carried out with the Superscript III Reverse Transcriptase kit (Invitrogen) according to manufacturer's instructions, using DNAse-treated RNA samples as a template. The integrity of the obtained cDNA samples was tested by amplification of the endogenous actin-β (*ACTB*) gene, and visualization by agarose gel electrophoresis. qPCR was carried out using SYBR green master mix (Applied Biosystems) and the ΔΔCt method (Livak and Schmittgen, 2001). Each reaction had a total volume of 15 μL, containing 5.4 μL of water, 7.5 μL of SYBR Green, 0.75 μL (10 μM stock) of each forward and reverse primers (manufactured at Integrated DNA Technologies, USA) and 0.6 μL of cDNA obtained from RT-PCR reactions, for each sample. The reactions were mounted in 96 wells polypropylene plates covered with microplate adhesives. The reactions were carried out in an Applied Biosystems 7500 Real-Time PCR System (Applied Biosystems, UK) using the primer sets *TIMP2*: forward 5' - TTC CCT CCC TCA AAG ACT GA - 3', reverse 5' - CGT CTG GCT AAT TGC ATC CT - 3'; *RHOA*: forward 5'- GAG TTG GCT TTG TGG GAC AC - 3', reverse 5' - ACT ATC AGG GCT GTC GAT GG - 3'; *LIMK2:* forward 5' - TGC ACA TCA GTC CCA ACA AT - 3', reverse 5' - CGT CTG GCT AAT TGC ATC CT - 3'; *ACTB*: forward 5'- TTG CCG ACA GGA TGC AGA AGG A - 3', reverse 5'- AGG TGG ACA GCG AGG CCA GGA T- 3', with an annealing temperature near 60 °C and an amplicon of 100-150 bp. PCR conditions were: 50 °C for 2 min, 10 min at 95 °C, followed by 40 cycles at 95 °C for 15 s, and at 60 °C for 60 s. Dissociation curves were set up as follows: 95 °C for 15 s, 60 °C for 20 s and 95 °C for 15 s.

###  Statistical analysis

Statistical analyses for survival and apoptosis induction assays were performed by using the Student's *t* test, and a value of p ≤ 0.05 was considered as significant.

## Results

**Figure 1:**
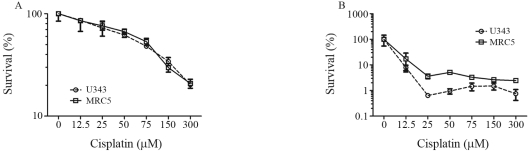
Cell survival. U343 and MRC-5 cell lines were treated with increasing concentrations of cisplatin (12.5; 25; 50; 75; 150 and 300 μM). The cells were harvested 24 h (A) and 5 days (B) after treatment (mean ± SD). Cell survival was measured by XTT assay.

The cytotoxic effect of cisplatin was analyzed in U343 and MRC-5 cells treated with different drug concentrations (12.5; 25; 50; 75; 150 and 300 μM). After 24 h of treatment (Figure 1A), cisplatin induced a 20 to 80% reduction in U343 cell survival, with a marked reduction in survival rates to less than 1% after 5 days of drug treatment (Figure 1B). MRC-5 cells also showed a reduction in survival fractions after 24 h of cisplatin treatment similarly as U343 cells (Figure 1A), although after 5 days (Figure 1B), this was only slight when compared to U343 cells.

**Figure 2:**
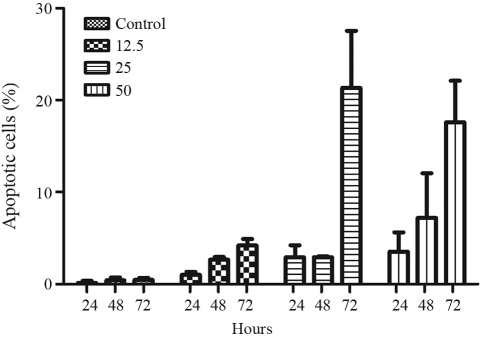
Frequency of apoptotic cells in U343 cell cultures treated with different concentrations of cisplatin (12.5; 25 and 50 μM). The results were obtained 24, 48 and 72 h after treatment. 500 cells were analyzed for each experiment (mean ± SD).

**Figure 3:**
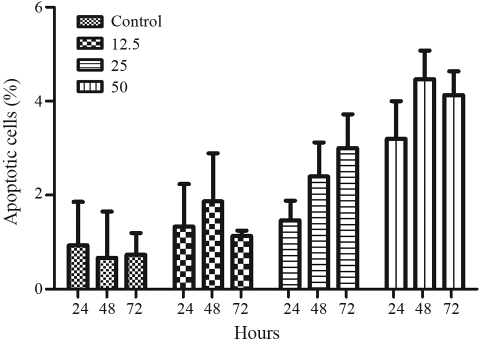
Frequency of apoptotic cells in MRC-5 cell cultures treated with different concentrations of cisplatin (12.5; 25 and 50 μM). The results were obtained 24, 48 and 72 h after treatment. 500 cells were analyzed for each experiment (mean ± SD).

Cisplatin-induced apoptosis occurred in U343 cells after treatment with 12.5, 25 and 50 μM for 24, 48 and 72 h (Figure 2). Analysis of cell morphology revealed apoptotic cells even after 24 h of treatment (3%), with higher frequencies after 48 (8%) and 72 h (20.4%) for 25 μM cisplatin. The apoptosis frequency displayed by MRC-5 cells after cisplatin treatment (Figure 3) was very low (4%). Thus, on the basis of these results, the U343 glioma cell line proved to be more sensitive to cisplatin than the normal fibroblast cell line (MRC-5) under similar conditions. On the contrary, the T98G glioma cell line was very resistant to cisplatin treatment at increasing concentrations (data not shown).

Alterations in gene expression were evaluated in U343 cells treated with 25 μM cisplatin, and RNA extraction was performed after 48 h. Statistical analysis was carried out by the SAM method, which indicated a total of 67 differentially expressed genes: 29 down-regulated and 38 up-regulated genes at a FDR < 0.05 (Table 1). Regarding to biological functions attributed to the set of significant genes, the most frequent categories (represented by a variable number of genes) were related to metabolism, ubiquitin-proteasome, cell proliferation, adhesion, apoptosis, cell cycle and DNA repair.

**Figure 4:**
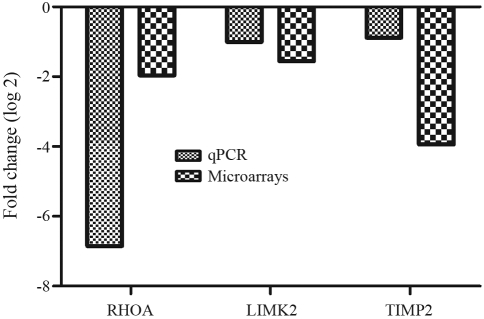
Gene expression levels determined by the cDNA microarray and real time PCR methods for *RHOA*, *LIMK* and TIMP2. The same RNA samples were used in both methods. The ΔΔct-values represent the log ratio (base 2).

By applying the real time PCR method, we confirmed the down-regulation of *RHOA*, *LIMK2* and *TIMP2* genes*,* by using the same remaining RNA samples as those employed in the microarray experiments. The results indicated similar gene expression patterns obtained by both methods (Figure 4). These genes were selected based on their functions associated with glioma cells. There were certain variations regarding the magnitude of relative expression, although gene expression modulation occurred in the same direction.

## Discussion

It is well known that cisplatin cytotoxicity is attributed to the formation of various DNA adducts that trigger cellular responses culminating in cell death (Zhang *et al.*, 2006). Studies on the quantitative and qualitative modulation of gene expression profiles under conditions of drug treatment is an interesting approach to characterize the mechanisms by which chemotherapeutic agents act on cancer cells. Although cisplatin has been used for a long time, the molecular mechanisms of cell responses associated to its cytotoxic activity are poorly clarified. In the present work, we first studied the potential of cisplatin to induce cell death in the glioma U343 cell line. When compared to the SV40 transformed fibroblast cell line (MRC-5), U343 cells proved to be the more sensitive to cisplatin.

Survival experiments carried out with increasing drug concentrations confirmed the high potential of cisplatin to induce cytotoxic effects, as well as apoptosis, in U343 cells. Furthermore, a strong residual cytotoxic effect could still be observed several days following drug treatment. Survival analysis performed after 5 days demonstrated a significant reduction in the survival rates following drug treatment (12.5 to 300 μM), and a pronounced effect was observed at concentrations higher than 25 μM. The analysis of apoptosis showed that 25 μM cisplatin induced 20.4% of apoptotic cells following 72 h, indicating that some considerable proportion of cells died by apoptosis. However, damaged cells can also be effectively eliminated by other processes, such as necrosis, mitotic catastrophe, autophagy, as well as premature senescence, which irreversibly arrests cell division (Brown and Attardi, 2005).

We also tested temozolomide against a panel of glioma cell lines, viz., U343, U87, U251, U138 and T98G, in the laboratory, and only T98G cells were found to be sensitive to various concentrations of temozolomide (data not shown). According to other authors, cisplatin decreased the viability of A172 glioma cells in a time- and dose-dependent manner. Furthermore, cisplatin induced cytotoxicity in A172 cells showed characteristics related to apoptosis (Park *et al.*, 2006). Apoptosis is a common response of cells to platinum compounds (Sorenson *et al.*, 1990), and accordingly, in the present study we observed apoptosis as the primary effect of cisplatin on glioma cells.

Evaluation of gene expression can provide information on regulatory mechanisms, biochemical pathways and potential targets for clinical intervention and therapies in a variety of diseases (Zhang *et al.*, 2006). The expression profiles of drug-treated cells can be readily compared with untreated control cells to reveal sets of genes that have undergone alterations at the transcriptional level in response to drug treatment (Duale *et al.*, 2007). In the present study, the findings concerning gene expression profiles disclosed 67 significantly modulated genes in U343 cells treated with 25 μM cisplatin for 48 h. The experimental conditions of drug treatment were chosen on the basis of results from survival and apoptosis experiments. The statistical analysis carried out by SAM was applied to identify those gene signatures whose mRNA levels were significantly and differentially expressed between cisplatin- treated and untreated U343 cells. The quantitative results of gene expression indicated a set of up- and down-regulated genes, mainly related to metabolism, ubiquitin-proteasome, cell proliferation, adhesion, apoptosis, cell cycle control and DNA repair. Among the exclusively modulated genes, only a few were selected for discussion, and this was mainly due to their biological relevance. In the case of three genes (*RHOA*, *LIMK2* and *TIMP2*), the expression pattern was confirmed by the real time PCR technique, and was compatible with the results obtained by the microarray method.

In the set of genes modulated by cisplatin, the most frequent category was related to metabolism, represented by two up-regulated (*FUT8* and *ULK2*) and six down-regulated genes (*COX4I1*, *DYRK3*, *TBCD*, *LIMK2*, *MOCS2* and *P4HB*).

Some of these, such as *DYRK3*, play a role in cell growth and development in the glioma cell line (Yamanaka *et al.*, 2006), whereas *LIMK2* is involved in stress fiber and focal adhesion formation and membrane blebs during the apoptotic process. The down-regulation of *LIMK2*, also demonstrated by the real time PCR method, may affect several functions, including apoptosis induction. In fibrosarcoma, the reduced expression in *LIMK2* protein was found to restrict the metastatic potential (Suyama *et al.*, 2004). Some modulated genes, such as *USP38* and *PSMA1*, were related to the proteasome system. The ubiquitin-proteasome system is responsible for the degradation of both damaged proteins and regulators of growth and stress response. Alterations in this proteolytic system are associated with various forms of human pathologies (Deng *et al.*, 2007). Ubiquitin specific proteases (USPs) belong to a complex family of deubiquitinating enzymes that specifically cleave ubiquitin conjugates in a great variety of substrates, thereby regulating the production and recycling of ubiquitin itself, and are critically involved in the control of cell growth, differentiation, and apoptosis (Ovaa *et al.*, 2004; Rolen *et al.*, 2006).

U343 cells treated with cisplatin also showed up-regulated (*ADAMTS1* and *CDH13*), and down-regulated genes (*GNB1*, *TIMP2*, *RHOA* and *ING1*) related to cell proliferation. *ADAMTS1* negatively regulates tumor growth and metastasis (Vazquez *et al.*, 1999; Luque *et al.*, 2003; Choi *et al.*, 2008) , whereas *TIMP2* takes part in degrading ECM (extracellular matrix) and regulating the invasion process (Lu *et al.*, 2004), considered the root cause of the high recurrent incidence in glioblastoma (Kong *et al.*, 2007). TIMPs have also been shown to exert pluripotential effects on cell growth, apoptosis and differentiation (Baker *et al.*, 2002; Jiang *et al.*, 2002). Similar to *TIMP2*, the *RHOA* gene was also down-regulated in cisplatin-treated glioma cells, and the decreased expression levels were also confirmed through real time PCR analysis. The protein encoded by *RHOA* is involved in cell proliferation/stress response, and belongs to the Rho GTPases family which participates in cell growth, lipid metabolism cytoarchitecture, membrane trafficking, transcriptional regulation and apoptosis in response to genotoxic agents. They trigger specific signals that lead to uncontrolled cell growth, enhanced angiogenesis, inhibition of apoptosis and genetic instability, thus resulting in tumor development (Aznar and Lacal, 2001; Lu *et al.*, 2009). In astrocytomas, *RHOA* expression positively correlates with the degree of malignancy (Yan *et al.*, 2006).

One of the most distinct features of gliomas is the invasive growth pattern, which prevents total surgical resection. Their ability to infiltrate into normal brain parenchyma is associated to the process of cellular adhesion (Giese *et al.*, 1994). In the present work, we found five modulated genes under cisplatin treatment, which are closely related to adhesion. Among these, *CDH13*, *TIAM1* and *PCDH17* were up- and *FLRT1* and *OPCML* down-regulated, thus indicating that the invasion capacity of glioma cells can be altered by cisplatin treatment.

*OPCML* is significantly down-regulated in brain tumors, including gliomas (Reed *et al.*, 2007). This is a stress-responsive and TP53-regulated gene, capable of acting as a broad tumor suppressor for multiple tumor types (Cui *et al.*, 2008). The protein encoded by this gene is an opioid-binding cell adhesion molecule, which is often found methylated in ovarian cancers (Sellar *et al.*, 2003). Tumor cell invasion involves complex interactions between normal and malignant cells. It is well established that this dynamic process requires the concerted effects of various molecules including proteolytic enzymes, growth factors, adhesion molecules and extracellular matrix molecules (Cui *et al.*, 2008).

Cell response to induced DNA damage is a highly complex event that is orchestrated by a multitude of proteins and signaling pathways operating together in a cell context to activate mechanisms of DNA repair, cell cycle arrest and apoptosis, all depending on the extent of the DNA damage. In the present work, we analyzed gene expression profiles under conditions of apoptosis induction by cisplatin in the U343 cell line. Several modulated genes were related to apoptotic cell death (*TNFRSF10B*, *BCL-XL*, *APIP*, *SEMA6A*, *CRADD* and *P2RX4*). These findings suggest that the altered expression pattern of apoptosis related genes caused by cisplatin may be involved in chemosensitivity, as observed in survival assaying and in the frequency of induced apoptosis. Duale *et al.* (2007) found several apoptosis related genes in testicular germ cell tumors after cisplatin exposure (including BCL-2 family genes), suggesting the sensitivity of these cell lines to chemotherapeutic agents.

Some other cisplatin-modulated genes were related to cell cycle control (*TBRG1*, *SEPT2*, *ING1* and *TUSC4*) and DNA repair (*RAD51C*). Septins are involved in several processes, including membrane dynamics, vesicle trafficking, apoptosis, infection and cytoskeletal remodeling (Hall *et al.*, 2005). SEPT2 is a cell cycle-regulated protein, essential for cytokinesis in human astrocytoma cells (Kim *et al.*, 2004). Kremer *et al.* (2007) demonstrated a link between septins, the actin cytoskeleton and DNA damage checkpoint response.

ING proteins play a significant role in several important cellular processes, such as growth regulation, senescence, apoptosis, DNA repair and cell migration (Ythier *et al.*, 2008; Shah *et al.*, 2009)). TP53 target genes such as *p21WAF1* and *BAX,* have previously been identified as downstream targets of *p33ING1* and *p32ING2* (isoforms of the ING family) (Feng *et al.*, 2006). LN229 glioblastoma cells differentially up-regulated *p47ING1a* in response to cisplatin, this possibly representing a protective response against drug-induced DNA damage (Tallen *et al.*, 2008).

The HRR (Homologous Recombination Repair) pathway is critically important in the repair of DNA damage induced by crosslink agents, such as cisplatin (Golding *et al.*, 2004; Jayathilaka *et al.*, 2008). However, only the *RAD51C* gene was induced in cisplatin-treated glioma cells, probably due to the high level of drug cytotoxicity at the conditions tested. *RAD51* plays a role in the strand invasion and exchange between a free DNA-end proximal to the damaged site and a homologous double stranded DNA (Kuznetsov *et al.*, 2009).

In U373 glioblastoma cells undergoing cisplatin treatment, several genes were modulated, including those encoding proteins involved in transcriptional regulation, stress response, signal transduction, metabolism, cell structure and adhesion, apoptosis and survival, inflammation and immune responses, and other processes (Ma *et al.*, 2006). Li *et al.* (2007) encountered altered expression in several genes involved in DNA repair, apoptosis, cell cycle control and metabolism in ovarian cancer cells that had been exposed to cisplatin for several hours, whereas Bassi *et al.* (2008) also came upon genes connected with DNA repair modulated in response to ionizing radiation in U343 glioma cells.

In conclusion, cisplatin-treated U343 cells showed transcriptional changes that reflect several biological processes that were affected in consequence of drug treatment. These processes are related to the extensive DNA damage caused by cisplatin treatment, visualized through the amount of induced cell death. These findings highlight the complexity of cellular responses and the signaling pathways ultimately leading to cell death in glioma cells.

## Figures and Tables

**Table 1:** Genes differentially expressed in the U343 glioma cell line after cisplatin treatment (25 μM for 48 h) selected through SAM analysis (FDR < 0.05).

Gene symbol	Unigene ID	Description	Fold-change	Function
LIMK2	Hs.474596	LIM domain kinase 2	-3.87	Metabolism
BCLXL	Hs.516966	BCL2-like 1	-2.48	Apoptosis
TIMP2	Hs.633514	Tissue inhibitor of metalloproteinase 2	-3.96	Cell Proliferation
VDP	Hs.292689	USO1 homolog, vesicle docking protein (yeast)	-3.53	Intracellular Transport
COG4	Hs.208680	Component of oligomeric golgi complex 4	-3.03	Protein Transport
RHOA	Hs.247077	Ras homolog gene family, member A	-2.95	Cell Proliferation
OPCML	Hs.4817	Opioid binding protein/cell adhesion molecule-like	-2.91	Adhesion
RQCD1	Hs.148767	RCD1 required for cell differentiation1 homolog (S. pombe)	-2.70	Transcription
ING1	Hs.46700	Inhibitor of growth family, member 1	-2.48	Cell Proliferation
FLRT1	Hs.584876	Fibronectin leucine rich transmembrane protein 1	-2.48	Adhesion
ING1	Hs.46700	Inhibitor of growth family, member 1	-2.48	Cell Cycle
COX4I1	Hs.433419	Cytochrome c oxidase subunit IV isoform 1	-2.11	Metabolism
GTF3C1	Hs.371718	General transcription factor IIIC, polypeptide 1, alpha 220kDa	-1.84	Transcription
TBCD	Hs.464391	Tubulin folding cofactor D	-1.79	Metabolism
CALU	Hs.7753	calumenin precursor	-1.69	Other Functions
MOCS2	Hs.163645	Molybdenum cofactor synthesis 2	-1.67	Metabolism
P4HB	Hs.464336	Procollagen-proline, 2-oxoglutarate 4-dioxygenase (proline 4-hydroxylase), beta polypeptide	-1.65	Metabolism
PASK	Hs.397891	PAS domain containing serine/threonine kinase	-1.59	Signal transduction
FZD4	Hs.591968	Frizzled homolog 4 (Drosophila)	-1.50	Other Functions
SPOP	Hs.463382	Speckle-type POZ protein	-1.37	RNA processing
CXCL10	Hs.632586	Chemokine (C-X-C motif) ligand 10	-1.24	Cell Signaling
NFKBIE	Hs.458276	Nuclear factor of kappa light polypeptide gene enhancer in B-cells inhibitor, epsilon	-1.13	Protein Localization
AP3S2	Hs.632161	Adaptor-related protein complex 3, sigma 2 subunit	-1.02	Protein Localization
DLX6	Hs.249196	Distal-less homeobox 6	-1.01	Regulation of Transcription
DYRK3	Hs.164267	Dual-specificity tyrosine-(Y)-phosphorylation regulated kinase 3	-0.90	Metabolism
TBRG1	Hs.436410	Transforming growth factor beta regulator 1	-0.72	Cell Cycle
AKAP7	Hs.486483	A kinase (PRKA) anchor protein 7	-0.71	RNA Metabolism
GNB1	Hs.430425	Guanine nucleotide binding protein (G protein), beta polypeptide 1	-0.69	Cell Proliferation
FUT8	Hs.654961	Fucosyltransferase 8 (alpha (1,6) fucosyltransferase)	0.10	Metabolism
PAPOLA	Hs.253726	Poly(A) polymerase alpha	0.11	Transcription
KCNV1	Hs.13285	Potassium channel, subfamily V, member 1	0.11	Ion Transport
CRADD	Hs.591016	CASP2 and RIPK1 domain containing adaptor with death domain	0.13	Apoptosis
ARPP21	Hs.475902	cyclic AMP-regulated phosphoprotein, 21 kD	0.13	Other Functions
SRPK2	Hs.285197	SFRS protein kinase 2	0.14	Cell Differentiation
KCNIP4	Hs.655705	Kv channel interacting protein 4	0.17	Ion Transport
BTF3	Hs.591768	Basic transcription factor 3	0.28	Transcription
TNFRSF1OB	Hs.521456	Tumor necrosis factor receptor superfamily, member 10b	0.47	Apoptosis
RGS4	Hs.386726	Regulator of G-protein signaling 4	0.47	Signal Transduction
CPLX2	Hs.193235	Complexin 2	0.64	Other Functions
EIF4G1	Hs.433750	Eukaryotic translation initiation factor 4 gamma, 1	1.07	RNA Metabolism
SEPT2	Hs.335057	Septin 2	1.33	Cell Cycle
IL10	Hs.193717	Interleukin 10	1,41	Immune Response
SFRS11	Hs.479693	Splicing factor, arginine/serine-rich 11	1.44	RNA Splicing
APIP	Hs.447794	APAF1 interacting protein	1.45	Apoptosis
ADAMTS1	Hs.643357	ADAM metallopeptidase with thrombospondin type 1 motif, 1	1.61	Cell Proliferation
TAF4	Hs.18857	TAF4 RNA polymerase II, TATA box binding protein (TBP)-associated factor, 135kDa	1.76	Regulation of Biological Process
ULK2	Hs.168762	Unc-51-like kinase 2 (C. elegans)	1.79	Metabolism
STAM	Hs.441498	Signal transducing adaptor molecule (SH3 domain and ITAM motif) 1	1.80	Signal Transduction
GPR108	Hs.167641	G protein-coupled receptor 108	1.80	Other Functions
MSX1	Hs.424414	Msh homeobox 1	1.97	Other Functions
RAB37	Hs.592097	RAB37, member RAS oncogene family	2.23	Signal Transduction
NFRKB	Hs.530539	Nuclear factor related to kappaB binding protein	2.52	Response to Stress
USP38	Hs.480848	Ubiquitin specific peptidase 38	2.63	Ubiquitin-Proteasome
PCDH17	Hs.106511	Protocadherin 17	2.79	Adhesion
MECP2	Hs.200716	Methyl CpG binding protein 2 (Rett syndrome)	2.82	Transcription
TIAM1	Hs.517228	T-cell lymphoma invasion and metastasis 1	2.92	Adhesion
TUSC4	Hs.437083	Tumor suppressor candidate 4	3.06	Cell Cycle
POLR2K	Hs.351475	Polymerase (RNA) II (DNA directed) polypeptide K, 7.0kDa	3.07	Transcription
PSMA1	Hs.102798	Proteasome (prosome, macropain) subunit, alpha type, 1	3.20	Ubiquitin-Proteasome
SEMA6A	Hs.156967	Sema domain, transmembrane domain (TM), and cytoplasmic domain, (semaphorin) 6A	3.35	Apoptosis
CDH13	Hs.654386	Cadherin 13, H-cadherin (heart)	3.38	Adhesion
NEK8	Hs.448468	NIMA (never in mitosis gene a)- related kinase 8	3.41	Other Functions
RAD51C	Hs.412587	RAD51 homolog C (S. cerevisiae)	3.73	DNA Repair
P2RX4	Hs.321709	Purinergic receptor P2X, ligand-gated ion channel, 4	3.80	Apoptosis
TNFAIP1	Hs.76090	Tumor necrosis factor, alpha-induced protein 1 (endothelial)	4.01	Immune Response
INSM1	Hs.89584	Insulinoma-associated 1	4.74	Cell differentiation
GTF3C4	Hs.656646	General transcription factor IIIC, polypeptide 4, 90kDa	5.01	Transcription
